# Ultrawidefield-to-Conventional Fundus Image Translation with Scaled Feature Registration and Distorted Vessel Correction

**DOI:** 10.3390/bioengineering12101046

**Published:** 2025-09-28

**Authors:** JuChan Kim, Junghyun Bum, Duc-Tai Le, Chang-Hwan Son, Eun Jung Lee, Jong Chul Han, Hyunseung Choo

**Affiliations:** 1 Department of AI Systems Engineering, Sungkyunkwan University, Suwon 16419, Republic of Korea; wncks0928@skku.edu; 2 Department of Electrical and Computer Engineering, Sungkyunkwan University, Suwon 16419, Republic of Korea; bumjh@skku.edu (J.B.);; 3 Department of Software Science and Engineering, Kunsan National University, Gunsan 54150, Republic of Korea; cson@kunsan.ac.kr; 4 Department of Ophthalmology, Samsung Medical Center, Seoul 06351, Republic of Korea; 5 School of Medicine, Sungkyunkwan University, Suwon 16419, Republic of Korea; 6 Samsung Advanced Institute for Health Sciences and Technology, Sungkyunkwan University, Suwon 16419, Republic of Korea

**Keywords:** fundus image, multi-modal imaging, paired-learning, image registration, image translation

## Abstract

Conventional fundus (CF) and ultrawidefield fundus (UF) imaging are two primary modalities widely used in ophthalmology. Despite the complementary use of both imaging modalities in clinical practice, existing research on fundus image translation has yet to reach clinical viability and often lacks the necessary accuracy and detail required for practical medical use. Additionally, collecting paired UFI-CFI data from the same patients presents significant limitations, and unpaired learning-based generative models frequently suffer from distortion phenomena, such as hallucinations. This study introduces an enhanced modality transformation method to improve the diagnostic support capabilities of deep learning models in ophthalmology. The proposed method translates UF images (UFIs) into CF images (CFIs), potentially replacing the dual-imaging approach commonly used in clinical practice. This replacement can significantly reduce financial and temporal burdens on patients. To achieve this, this study leveraged UFI–CFI image pairs obtained from the same patient on the same day. This approach minimizes information distortion and accurately converts the two modalities. Our model employs scaled feature registration and distorted vessel correction methods to align UFI–CFI pairs effectively. The generated CFIs not only enhance image quality and better represent the retinal area compared to existing methods but also effectively preserve disease-related details from UFIs, aiding in accurate diagnosis. Furthermore, compared with existing methods, our model demonstrated a substantial 18.2% reduction in MSE, a 7.2% increase in PSNR, and a 12.7% improvement in SSIM metrics. Notably, our results show that the generated CFIs are nearly indistinguishable from the real CFIs, as confirmed by ophthalmologists.

## 1. Introduction

**Figure 1:**
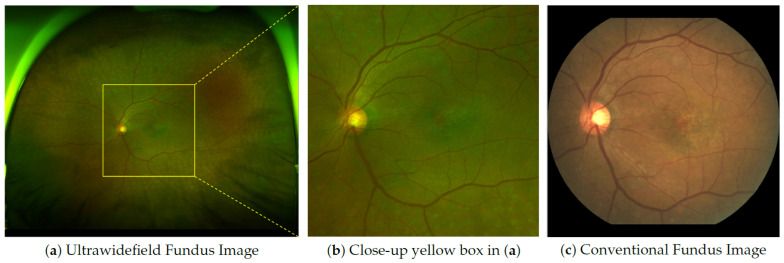
Sample of Ultrawidefield Fundus Image (UFI) and Conventional Fundus Image (CFI) pair. (**a**) UFI. (**b**) Close-up view of the yellow box in (**a**). (**c**) CFI.

Fundus imaging plays a crucial role in diagnosing and monitoring various pathological conditions. Conventional fundus (CF) imaging and ultra-widefield (UF) fundus imaging are the two major modalities widely used in clinical practice. Although CF imaging offers a limited field of view (∼45°) and captures only ∼15% of the retinal area, it remains the golden standard for diagnosing pathological conditions. Its long-standing clinical adoption by ophthalmologists is due to its effectiveness and reliability in diagnosing specific retinal diseases such as age-related macular degeneration (AMD), diabetic retinopathy, and glaucoma. These diseases commonly affect areas of the macula and optic disc, where photoreceptor cells are densely concentrated [1,2]. Furthermore, CF imaging involves dilating the patient’s pupil using a mydriatic agent and capturing visible-light images of the fundus. This procedure leads to temporary visual impairment that lasts for 2–3 h. Recently, UF imaging has emerged as a promising method that uses a non-mydriatic imaging method to minimize the physical burden on patients. UF imaging offers a field-of-view of approximately 200°, which covers up to 80% of the entire retinal area [3]. Consequently, UF imaging is particularly useful for the early detection of conditions such as drusen and epiretinal membranes (ERM), which are more prevalent in expansive areas of the retina [1,3]. UFIs encompass the area of CF images (CFIs); therefore, information on CFIs should ideally be visible in the UFIs, as shown in Figure 1. However, because of the limitations of UF imaging, UFIs exhibit image distortion and relatively low image quality in the macular and optic disc areas. Consequently, relying solely on UFI to diagnose ophthalmic diseases is challenging. Ophthalmologists often perform UF and CF imaging simultaneously to ensure an accurate diagnosis. However, this practice can impose additional financial, time, and medical resource burdens on patients [4].

To address these challenges, this study proposes translating UFIs into CFIs to reduce the need for multiple imaging sessions and improve diagnostic accessibility. Prior approaches attempted UFI-to-CFI translation using conventional computer vision methods, such as cropping and resizing [4,5]. These techniques extract the central regions from UFIs that approximately correspond to the CFI field of view. However, owing to substantial differences in scale, resolution, and color spectrum between the two modalities, largely caused by differences in light transmission and mosaicking artifacts, the resulting macular and optic disc visibility is compromised and lacks reliability for clinical interpretation [6].

Recent advances have shifted the focus toward deep-learning-based translation. Early studies employed unpaired learning frameworks such as CycleGAN [7], which enabled image-to-image translation without requiring matched input-output pairs. To support training, these models apply intensity-based registration methods to align the UFIs into CFI-like shapes using predefined templates [1,8]. However, mismatched templates, contrast inconsistencies, and brightness variabilities, particularly in atypical UFIs, often result in suboptimal outcomes. Attention-based models [9] incorporating dual-illumination correction [10] and the convolutional block attention module (CBAM) [11] have introduced modest improvements by emphasizing anatomical features such as the optic disc and retinal vessels. Additional preprocessing using a Faster R-CNN for optic disc localization [12] was also explored, although the robustness and consistency remained insufficient for practical deployment.

In parallel, diffusion-based and transformer-augmented frameworks have been introduced for fundus image synthesis and cross-modality translation. For example, ultra-widefield-to-angiography translation has been explored using diffusion models [13,14] and ControlNet-style conditioning [15], which can generate anatomically realistic content and enhance the global image characteristics. However, these methods cannot reproduce fine-scale structures, such as capillaries or subtle lesions, such as drusen, exhibit greater brightness variability, and require large high-quality datasets [13,14,15,16,17]. Transformer-based architectures, which are widely used for fundus analysis tasks, including classification and image quality assessment [18,19,20], offer long-range context modeling, but have not been adapted for controllable, paired cross-modality translation [21]. Although these developments are highly relevant, a direct comparison of diffusion- or transformer-based approaches will be addressed in future work. This study focuses on a registration-centric paired-learning framework as a data-efficient baseline, designed to achieve reliable UFI-to-CFI translation while preserving anatomical fidelity and fine-detail structures [1,17,21].

UFIs and CFIs from unrelated patients are arbitrarily paired in unpaired learning, making translation prone to clinically irrelevant structures and structural distortion owing to domain mismatch and inter-patient variability [1,9]. However, these limitations significantly hinder their clinical utility. Paired learning frameworks attempt to mitigate these issues by training on one-to-one UFI–CFI pairs. This approach provides consistent supervision and reduces the ambiguity during translation [6,22]. However, such methods are underutilized because of the limited availability of paired datasets. Even with paired data, substantial differences in scale, resolution, and field of view between UFIs and CFIs introduce alignment challenges and translation artifacts. As UFIs are often synthesized from multiple frames, geometric distortions and overlapping or duplicated anatomical regions may result in information loss or incorrect mapping when directly translated into the CFI domain.

These challenges highlight the need for enhanced multimodal registration techniques to align the UFIs and CFIs within a unified spatial framework. Traditional fundus image registration methods include intensity-based techniques that maximize pixel-wise similarity metrics using affine or deformable transformations [23,24] and feature-based techniques that detect and match anatomical landmarks using handcrafted descriptors [25,26,27]. However, both approaches exhibit limited performance when applied to cross-modality registration involving large-scale disparities such as between 200° UFIs and 45° CFIs. To improve intermodality alignment, some studies have attempted to segment the optic disc (OD) region and apply geometric rescaling before registration. Although this strategy offers localized structural guidance, its effectiveness remains limited owing to inconsistencies in OD localization and variability in image quality across samples [28,29]. More recently, deep-learning-based registration methods have been developed to estimate spatial transformations in an end-to-end manner [30,31,32]; however, most have been optimized for same-modality images and tend to perform poorly when confronted with large domain shifts [27].

To the best of our knowledge, UFI-to-CFI translation via paired learning has been underexplored owing to the scarcity of paired datasets and lack of thorough clinical evaluations. For clinical applicability, models must be trained using same-day UFI–CFI pairs from the same patient to minimize distortions due to physiological changes or viewpoint differences and for precise mapping of the lesion. Therefore, we propose a novel UFI-to-CFI paired-learning translation framework that integrates scale feature registration and distorted-vessel correction. Scale-feature registration improves the focus on relevant regions by aligning key anatomical landmarks, whereas distorted-vessel correction mitigates the vessel deformation inherent in UFIs, thereby enhancing translation fidelity [33]. Existing unpaired methods fall short of simultaneously addressing both challenges, thus motivating the design of a more robust solution.

A comparative analysis with previous work, particularly the methodology proposed by Pham et al. [9], reveals that our framework exhibits superior performance across both quantitative and qualitative evaluation criteria. Quantitatively, our method yields an 18.2% reduction in the mean squared error (MSE), a 7.2% enhancement in the peak signal-to-noise ratio (PSNR), and a 12.7% improvement in the structural similarity index measure (SSIM) metrics relative to existing approaches. These empirical findings are further substantiated by independent qualitative assessments conducted by clinical ophthalmologists. This validates the robustness and clinical relevance of the proposed methodology. The enhanced image fidelity achieved using our approach contributes to improved diagnostic accuracy in retinal pathology detection, while potentially mitigating the clinical burden associated with multiple imaging modalities [34]. Comprehensive algorithmic implementation details and systematic performance evaluations are described in subsequent sections.

## 2. Materials and Methods

### 2.1. Study Cohort

In this section, we retrospectively collected retinal image pairs from patients with ocular diseases at Samsung Medical Center between January 2010 and November 2021. Patients who underwent both UFI and CFI on the same day were selected to ensure temporal consistency. UFIs were acquired using the Optos Daytona system (≈20 μm optical resolution and 200° field of view), and CFIs were acquired using a Topcon TRC-50DX camera (Topcon Corp., Tokyo, Japan). After excluding images with motion artifacts or of poor quality, 3578 high-quality image pairs were obtained from 2000 patients. Patient personal identifiable information was removed from all images before the analysis. All images were resized to 512 × 512 pixels during preprocessing. The dataset was split on a patient-independent basis into 2978 pairs for training, 300 for validation, and 300 for testing. For qualitative evaluation, 100 test pairs were independently reviewed by two board-certified glaucoma specialists who evaluated the clinical plausibility of the key diagnostic features. Disagreements were resolved through direct discussion. Because the task focused on image-to-image translation rather than diagnosis, no additional labels were included. This retrospective study complied with the principles of the Declaration of Helsinki and was approved by the Institutional Review Board of Samsung Medical Center (IRB no. SMC 2022-06-032), with a waiver of informed consent.

### 2.2. Overall Structure of the Proposed Method

The proposed aims to accurately translate the CFI from the UFI while minimizing distortions and information loss in critical regions, such as the macula, OD, and vascular structures. Although UFIs encompass the same anatomical regions as CFIs, their lower resolution often leads to deficient detail in these areas. To address this issue, the proposed method combines two strategies. (1) Scaled feature registration recalibrates the UFI by accounting for scale disparities between UFI and CFI, aligning vessels and OD to reduce the semantic gap and simplify the transformation task of the model. (2) The distorted vessel correction branch derives an edge map from the UFI that highlights CFI-relevant structural details (vessels, OD, and small pathological markers). The model elucidated these features with heightened clarity by incorporating a map as an auxiliary input. Clinically, this approach generates high-fidelity CFIs that preserve diagnostic information—facilitating early detection of macular pathology and vascular abnormalities, reduce the need for repeat imaging, and streamline the ophthalmic workflow.

**Figure 2:**
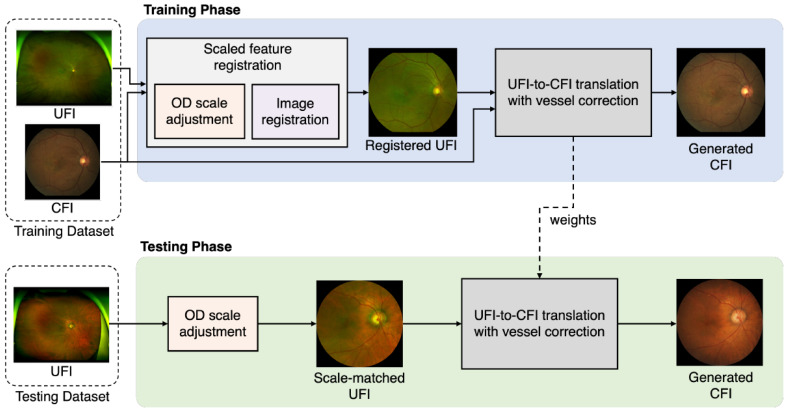
Overview of the proposed method. The framework consists of two phases: training and testing. During training, paired UFIs and CFIs are used to align spatial features via scaled registration, and a translation network is optimized to generate CFIs with corrected vessel structures. During testing, only a UFI is input; the model uses OD-based scale adjustment and the trained translation network to synthesize the corresponding CFI.

The proposed method consists of two phases—training and testing—as illustrated in Figure 2. Paired UFIs and CFIs from the same patients were used during training. First, scaled feature registration aligns the UFI with the CFI’s spatial dimensions and coordinate schema to produce a registered UFI. Next, the distorted vessel correction leveraged the registered UFI along with the real CFI to guide the optimization of the image translation model, yielding a representative CFI. In the testing phase, only the UFI is input, an OD scale adjustment identifies the region corresponding to the CFI field of view, and the distorted vessel correction branch extrapolates the final CFI using the trained parameters. Thus, model optimization relies on registered image pairs during training, whereas CFI synthesis during testing depends solely on the scale-matched UFI.

### 2.3. Scaled Feature Registration

**Figure 3:**
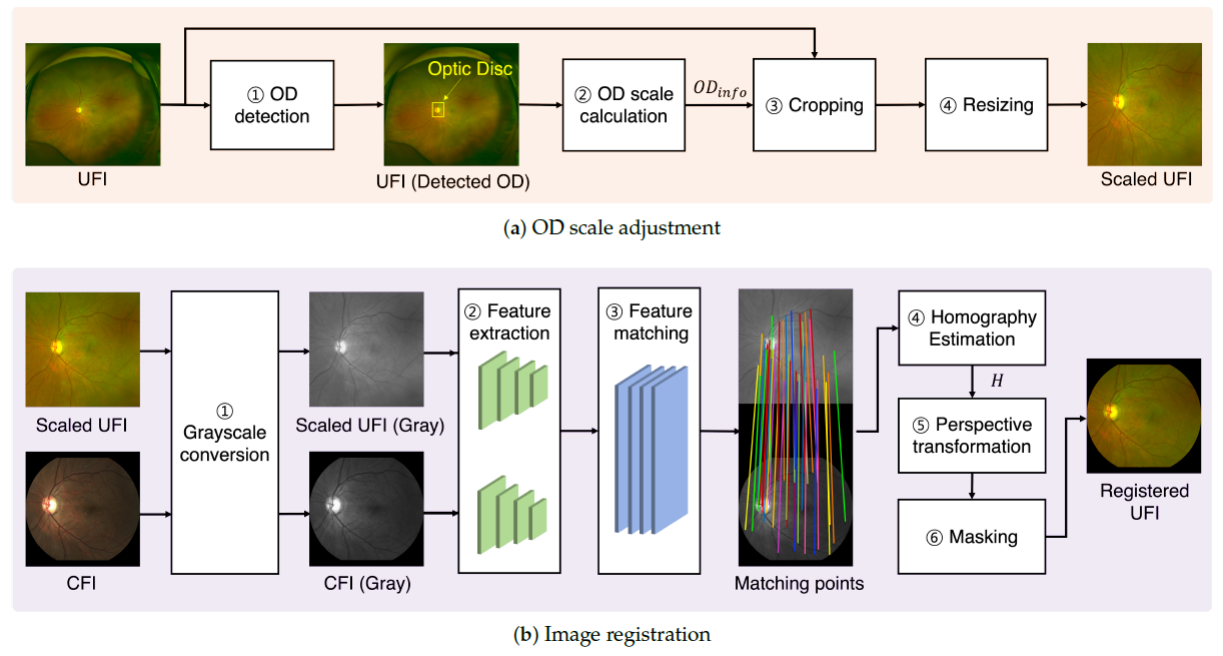
Two steps of scaled feature registration. (**a**) OD scale adjustment: The UFI is aligned to the CFI’s spatial scale by detecting the optic disc, computing an OD information vector ${\mathrm{OD}}_{\mathrm{info}}$, cropping the region of interest, and resizing the image to the target resolution. (**b**) Image registration: The scaled UFI and the CFI are converted to grayscale, followed by feature extraction and matching. A homography matrix is computed from matched keypoints, which is then used for perspective transformation and masking to produce a spatially registered UFI.

The proposed image-translation pipeline employs a two-step registration framework to align UFI with CFI, addressing both spatial-scale discrepancies and geometric misalignments. The overall workflow is illustrated in Figure 3.

Step 1: Optic Disc (OD) Scale Adjustment aligns the unscaled UFI ($I_{U}$) to the spatial resolution of the CFI using the optic disc (OD) as a stable anatomical reference. This process ensured spatial normalization before image registration and translation. It consists of the following subcomponents: (i) OD Localization: A pretrained Faster R-CNN model is used to detect the OD in the UFI, outputting a bounding box that encodes its approximate position and size. (ii) Region Information Extraction From this bounding box, an OD information vector ${\mathrm{OD}}_{\mathrm{info}}$ is computed, which captures the relative coordinates and scale of the OD normalized to [0,1]. (iii) Cropping Using ${\mathrm{OD}}_{\mathrm{info}}$, the UFI is cropped to center the OD with a crop window set to six times the width and height of the OD. (iv) Resizing: the cropped image is resized to match the target CFI resolution for consistent spatial dimensions.

This sequence of operations is formalized as follows.

(1) $$\begin{array}{cc} {\mathrm{OD}}_{\mathrm{info}} & =\mathrm{ExtractOD}(\mathrm{LocalizeOD}\left(I_{U}\right)) \end{array}$$

where $I_{U}$ is the input UFI. LocalizeOD(·) localizes the optic disc in $I_{U}$ using a pretrained detector, and ExtractOD(·) derive the normalized coordinates and size information from the detected bounding box.

(2) $$\begin{array}{c} I_{U}^{*}=\mathrm{Align}\left(\mathrm{Crop}\left(I_{U},{\mathrm{OD}}_{\mathrm{info}}\right),l\right) \end{array}$$

where Crop(·) centers and crops the UFI based on the OD location and scale extracted from ${\mathrm{OD}}_{\mathrm{info}}$. Align(·) resizes the cropped image to match the target CFI resolution *l*. $I_{U}^{*}$ denotes the final scale-adjusted UFI used for the downstream translation.

Step 2: Feature-Based Image Registration aligns the scale-normalized UFI ($I_{U}^{*}$) to the target CFI ($I_{C}$) using a feature-based approach to ensure spatial correspondence. This process ensures accurate spatial alignment, which is critical for downstream translation. It consists of the following subcomponents: (i) Grayscale Conversion: Both $I_{U}^{*}$ and $I_{C}$ are converted to grayscale to enhance structural similarity and mitigate color inconsistencies for feature detection. (ii) Feature Extraction: Feature descriptors are extracted from both images using a neural network trained on retinal landmarks; (iii) Feature Matching: The corresponding feature points are matched using a graph-based matcher (e.g., SuperGlue) that improves robustness by leveraging the spatial context. (iv) Homography Estimation: A homography matrix *H* is computed from the matched feature pairs, modeling the transformation from $I_{U}^{*}$ to $I_{C}$; (v) Perspective Transformation—The UFI is warped using a homography matrix to align it with the CFI’s geometry. (vi) Masking—A binary mask is applied to retain only the valid overlapping regions and suppress irrelevant areas.

This sequence of operations is formalized as follows.

(3) $$\left[P_{S},P_{T}\right]=\mathrm{Match}\left(\mathrm{FeatExt}\left(\mathrm{Gray}\left(I_{U}^{*},I_{C}\right)\right)\right),$$

where $I_{U}^{*}$ is the scaled UFI and $I_{C}$ is the target CFI. Gray(·) converts both images to grayscale, FeatExt(·) extracts feature descriptors, and Match(·) identifies keypoint correspondences, resulting in matched pairs $\left[P_{S},P_{T}\right]$.

(4) $$I_{U}^{**}=\mathrm{Mask}\left(\mathrm{Warp}\left(I_{U}^{*},\mathrm{HomoEst}\left(\left[P_{S},P_{T}\right]\right)\right)\right),$$

where HomoEst(·) estimates the homography matrix *H* from matched keypoints. Warp(·) applies the geometric transformation to $I_{U}^{*}$ using *H*, and Mask(·) filters the result to retain only valid overlapping regions, thereby producing the final aligned output $I_{U}^{**}$.

The homographic transformation is defined as follows:

(5) $$\left[\begin{array}{c} u^{\prime }\\ v^{\prime }\\ 1 \end{array}\right]=H\left[\begin{array}{c} u\\ v\\ 1 \end{array}\right]\;\mathrm{or}\;P_{T}=HP_{S},$$

where (u,v) and $\left(u^{\prime },v^{\prime }\right)$ denote the corresponding keypoints for $I_{U}^{*}$ and $I_{C}$, respectively. Matrix $H\in R^{3\times 3}$ encapsulates the perspective transformation between the two coordinate systems. Although a minimum of four-point correspondence is mathematically sufficient, a larger number of high-quality matches generally improves the robustness and accuracy of the estimated transformation [35].

### 2.4. UFI-to-CFI Translation with Distorted Vessel Correction Branch

**Figure 4:**
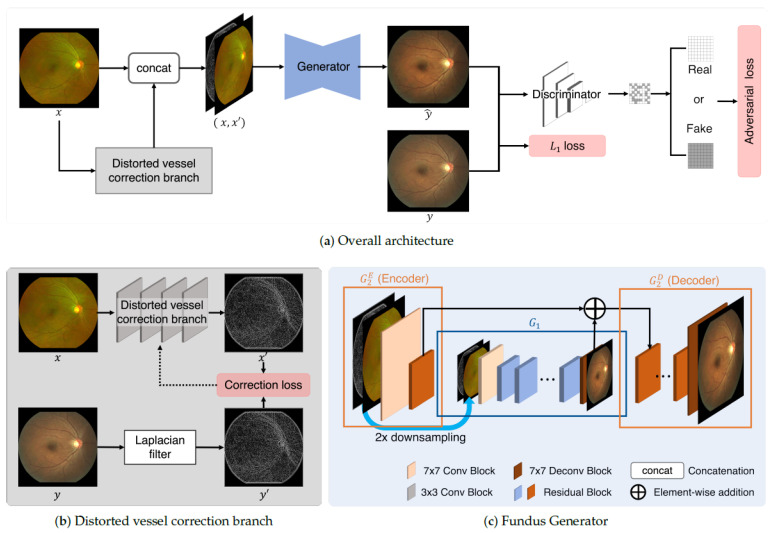
Architecture of the proposed UFI-to-CFI translation model. The framework consists of three main components: (**a**) Overall architecture, where the input UFI *x* is translated into CFI $\hat{y}$ by the generator and supervised with adversarial loss, $L_{1}$ reconstruction loss, and correction loss; (**b**) distorted vessel correction branch, which estimates the vessel edge map $x^{\prime }$ from the input *x* and is trained with correction loss against the Laplacian-filtered target CFI $y^{\prime }$; (**c**) fundus generator, composed of two subnetworks $G_{1}$ and $G_{2}$, where $G_{1}$ processes downsampled inputs and $G_{2}$ handles original-scale inputs with edge maps, combining both through residual and skip connections to ensure anatomically consistent and high-fidelity translation.

The proposed model is predicted on a pixel-to-pixel image-translation GAN [6,22] and aims to execute domain translation and image enhancement for registered UFI-CFI pairs. As illustrated in Figure 4, the proposed model converts the UFI domain into the CFI domain. It compares pixel values from the given registered UFI-CFI pairs corresponding to the same location and is trained to minimize the discrepancy between them. The distorted vessel correction model can be subdivided into three main components: the distorted vessel correction branch, the generator, and the discriminator. Here, the registered UFI input is represented by *x* and the CFI target is represented by *y* and $\hat{y}$.

The distorted-vessel correction branch is a shallow network composed of four convolutional blocks. It receives the registered UFI *x* as an input and predicts the edge map $x^{\prime }$, which approximates the vessel structure of the target CFI. The target edge map was derived by applying a Laplacian filter to the real CFI *y* for supervision during training. The inferred edge map $x^{\prime }$ was then concatenated with the original UFI and fed into the generator as a vessel-guided input.

The generator comprises two networks: $G_{1}$, which captures global features from a downsampled input, and $G_{2}$, which focuses on fine-grained details at the original resolution. Both $G_{1}$ and $G_{2}$ consist of 2× downsampling and upsampling convolutional layers at the input and output, respectively, with 16 residual blocks between them. The UFI is downsampled to half its original size and passed to $G_{1}$, whereas the full-resolution UFI and edge map are provided to $G_{2}$. The outputs from $G_{1}$ and the encoder of $G_{2}$ ($G_{2}^{E}$) are merged via skip connections and passed to the decoder of $G_{2}$ ($G_{2}^{D}$) to generate synthetic CFI $\hat{y}$.

The correction branch enhances translation accuracy by explicitly embedding the vessel structure information into the generator input. This is particularly beneficial in low-resolution UFI regions where vascular boundaries, such as the optic disc (OD) or macula may appear distorted. By learning to match the Laplacian-filtered CFI edges, the branch provides vessel-aware priors that guide the generator toward a more accurate synthesis.

During training, the generator learns to minimize pixel-level differences with the real CFI using $L_{1}$ loss, while also competing against the discriminator via adversarial loss. The discriminator adopts a PatchGAN [22] architecture, dividing the image into 7×7 patches and classifying each as either real or fake. This design enables the generator to produce realistic textures across local regions.

Traditional pixel-based loss functions often emphasize global color similarity, which can lead to blurred results and neglect fine structures. In contrast, the proposed model introduces a correction loss based on edge-level supervision, enabling it to preserve vascular details and improve anatomical fidelity in the generated outputs.

The generator generates the CFI $\hat{y}$ from the input UFI *x* using the edge-aware guidance $x^{\prime }$. The transformation process is defined as follows.

(6) $$\hat{y}=G_{2}^{D}\left(G_{2}^{E}\left(x,x^{\prime }\right)+G_{1}\left(\mathrm{Down}\left(x,x^{\prime }\right)\right)\right),$$

where *x* denotes the input UFI, $x^{\prime }$ is the edge map predicted by the distorted vessel correction branch from *x*, and Down(·) represents a 2× downsampling operation. $G_{1}$ is the global feature extractor applied to the downsampled input, and $G_{2}^{E}$ and $G_{2}^{D}$ are the encoder and decoder components of the local generator $G_{2}$, respectively. Output $\hat{y}$ is the generated CFI.

In this framework, the edge map $x^{\prime }$ is concatenated with the original input *x* and passed to both $G_{1}$ and $G_{2}^{E}$, whereas $G_{1}$ receives the downsampled version Down(x). The output features from $G_{1}$ and the encoded representation from $G_{2}^{E}$ are fused via element-wise addition and forwarded to the decoder $G_{2}^{D}$.

This hierarchical structure enabled the generator to integrate global contextual information with fine-grained anatomical details, thereby producing more realistic and structurally consistent CFI outputs.

Notably, $G_{2}$ consists of encoder $G_{2}^{E}$ and decoder $G_{2}^{D}$. The output feature maps from $G_{1}$ and the early residual layers of $G_{2}$ shared the same spatial dimensions and were merged via a skip connection, which was then passed to $G_{2}^{D}$. As illustrated in Figure 4, this design allows the generator to leverage both the global features from $G_{1}$ and the local edge-aware features from $G_{2}^{E}$ to reconstruct the final output via $G_{2}^{D}$. During training, the generator synthesizes a fake CFI $\hat{y}$ from a given UFI *x* and edge map $x^{\prime }$ and learns the transformation by comparing $\hat{y}$ with the real CFI *y*.

### 2.5. Loss Functions

To guide this translation process, we adopted a conditional generative adversarial network (cGAN) framework [8] that introduces input-dependent conditions into the standard GAN architecture. This enables the generator to produce images that not only follow the distribution of real CFIs but also remain structurally consistent with the input UFIs. The cGAN loss is defined as follows:

(7) $$L_{\mathrm{cGAN}}\left(G,D\right)=E_{x,y}\left[\log D\left(x,y\right)\right]+E_{x}\left[\log\left(1-D\left(x,G\left(x\right)\right)\right)\right],$$

where *x* and *y* denote the input UFI and target CFI, respectively, *G* is the generator, and *D* is the discriminator. Generator *G* is trained to minimize this loss by generating outputs that the discriminator classifies as real, whereas discriminator *D* is simultaneously trained to distinguish between the real and generated pairs. This adversarial setup allows both networks to iteratively improve during training.

However, owing to the generative nature of GANs, which focuses on distribution-level matching, the model may overlook fine anatomical details during translation. To avoid this, we introduce the additional losses described in the next section to preserve structural fidelity and enhance local feature realism.

To preserve the structural consistency between the generated and real CFIs, we introduce an $L_{1}$ reconstruction loss that penalizes the pixelwise differences between the two images:

(8) $$L_{1}=\frac{1}{N}\sum_{i=1}^{N}\left|y_{i}-G{\left(x\right)}_{i}\right|,$$

where $y_{i}$ is the *i*-th pixel of the real CFI and $G{\left(x\right)}_{i}$ is the corresponding pixel of the generated CFI. Although $L_{2}$ loss is often used for regression tasks, it produces overly smooth outputs in image synthesis. By contrast, $L_{1}$ loss function is less sensitive to outliers and better preserves sharp anatomical boundaries, which are essential for fundus image translation.

To further enhance the vessel structure estimation in the generator, we employed a vessel correction loss that supervises the distorted vessel correction branch. This branch is trained to predict an edge map that approximates the Laplacian-filtered version of the target CFI. Correction loss is defined as follows:

(9) $$L_{\mathrm{corr}}=\frac{1}{N}\sum_{i=1}^{N}{∥h{\left(y\right)}_{i}-F{\left(x\right)}_{i}∥}_{2},$$

where h(·) denotes a Laplacian filter applied to the real CFI *y* and F(·) is the distorted vessel correction branch that takes the UFI *x* as input. This loss encourages the model to extract vessel-edge features that are not readily visible in the UFI but are critical for accurate translation into the CFI domain.

The total loss function is expressed as follows:

(10) $$L=L_{\mathrm{cGAN}}+\lambda_{1}\;L_{1}+\lambda_{2}\;L_{\mathrm{corr}},$$

where $\lambda_{1}$ and $\lambda_{2}$ are the weights for the L1 and $L_{c}orr$ losses, respectively. In the training phase, the cGAN loss instructs the UFI, when input to the generator, to learn conditional to the general distribution of the Real CFI. Concurrently, the L1 and $L_{corr}$ losses ensure that the input image maintains its structural detail. During this process, adjusting the value of $\lambda_{1}$ is vital for preventing the discriminator from yielding to mode collapse. Relying solely on L1 loss inherently leads to blurry images; thus, we integrated both the cGAN loss and correction loss.

### 2.6. Training Models

The model training was performed using a global–local generator architecture [6] with a gradient branch. The backbone network employs a U-Net encoder-decoder structure. For scaled feature registration, we utilized modified SuperGlue model weights from the official GitHub repository (SuperGluePretrainedNetwork, Magicleap) [35] repository to enhance spatial alignment capabilities. The training utilized paired UFI–CFI samples resized to 512×512 pixels with a batch size of 64 and instance normalization. Experiments were conducted using a dual-Tesla A6000 GPUs (NVIDIA Corp., Santa Clara, CA, USA). The Adam optimizer with $\beta_{1}=0.5$ was employed at a learning rate of 0.0002. The training proceeded for 200 epochs with a fixed learning rate for the first 100 epochs, followed by a linear decay over the remaining 100 epochs. The global and local paths of the generator used 64 initial filters with nine and three residual blocks, respectively. A 3-layer PatchGAN discriminator was employed to enhance training stability and output quality, implemented in PyTorch.

## 3. Results

### 3.1. Evaluation of UFI-to-CFI Registration

**Figure 5:**
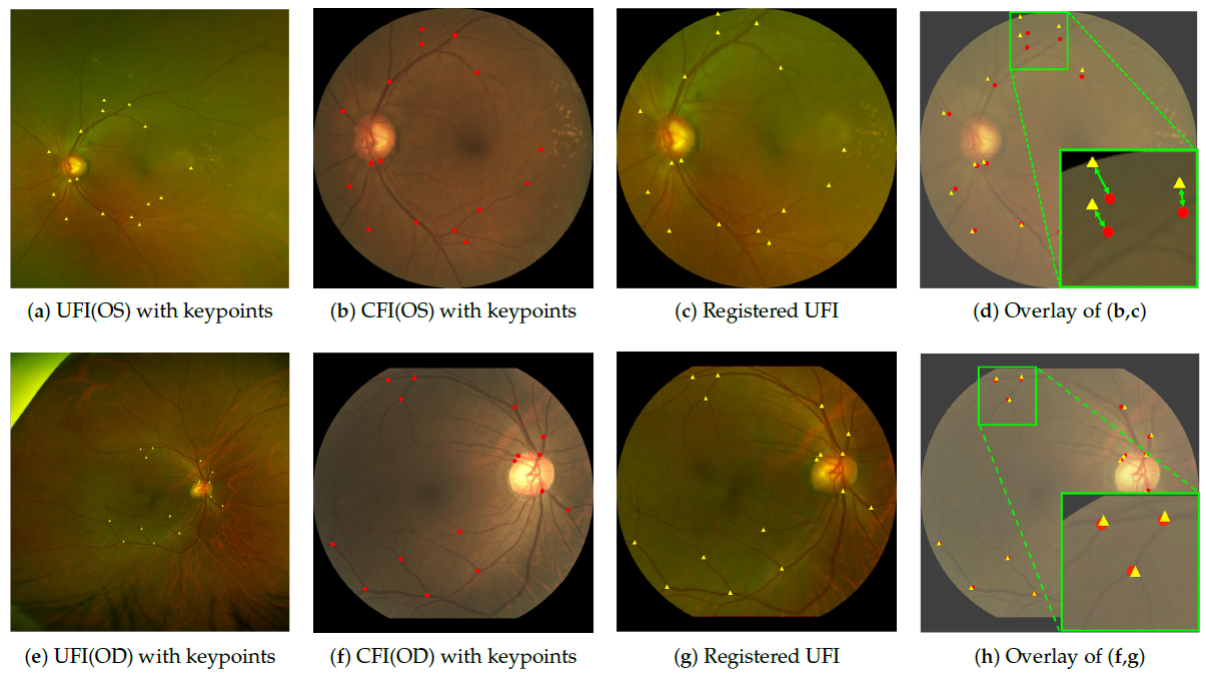
Visualization of labeled key points on CFI and registered UFI. The first row (subfigures (**a**–**d**)) corresponds to the left eye (oculus sinister, OS), and the second row (subfigures (**e**–**h**)) corresponds to the right eye (oculus dexter, OD). (**a**,**e**) are UFI; (**b**,**f**) are CFIs; (**c**,**g**) are the registration results of the proposed UFI-to-CFI registration method; and (**d**,**h**) are the overlap images of (**b**,**c**) and (**f**,**g**), respectively. The red dot indicates a key point of CFI, and the yellow triangle indicates a key point of UFI. The distance between the red dot and the yellow triangle in (**d**,**h**) indicates the registration performance: the closer the distance, the better the registration.

In this section, we quantitatively evaluated the quality of the UFI-to-CFI alignment using the percentage of correct keypoints (PCK) [36]. PCK measures the ratio of matched keypoints from the registered UFI and CFI that fall within a predefined spatial threshold, reflecting the geometric consistency between the two images. A higher PCK indicates better registration accuracy. To compute PCK, keypoints were manually annotated on common anatomical landmarks shared by both modalities, including the optic disc (OD) boundaries and vascular bifurcations. In total, 300 UFI–CFI pairs were labeled, with each pair containing 8–24 matched points, depending on the available anatomy. Figure 5a–c,e–g show examples of annotated keypoints, where the red and yellow circles denote points on the CFI and UFI, respectively.

For registration, both the UFI and its corresponding keypoints were transformed using the same homography matrix to align them with the target CFI. The alignment accuracy was visualized by overlaying the registered UFI and CFI keypoints, as shown in Figure 5d,h. In the magnified view, the distance between the matched keypoints serves as a visual indicator of registration quality. Notably, the first row of Figure 5d shows larger spatial discrepancies between the red and yellow triangles than the second row, indicating suboptimal alignment that results in local structural distortion, particularly in nearby blood vessels. These observations support the utility of PCK as a robust metric; well-aligned keypoints yield high PCK values and preserve anatomical consistency, whereas larger mismatches correspond to poor registration performance.

The percentage of correct keypoints (PCK) measures registration accuracy using manually labeled correspondences.

(11) $$\mathrm{PCK}=\frac{1}{n}\left|\left\{P_{S}^{i}\in P_{S}\;|\;d\left(\phi \left(P_{S}^{i}\right),P_{T}^{i}\right)<\tau \right\}\right|,$$

where $P_{S}^{i}$ and $P_{T}^{i}$ denote the *i*-th keypoints in the source (UFI) and target (CFI) images, respectively; ϕ is the registration transformation; and d(·,·) is the Euclidean distance. The threshold τ determines whether a keypoint match is considered correct and is defined as follows:

(12) τ=θr,

where *r* is the average optic disc (OD) radius in the dataset and θ is an adjustable coefficient that controls the strictness.

Table 1 presents the results of the quantitative PCK evaluation. It compares the performance of feature-based registration methods [35,37,38] before and after applying the scaled feature registration method. The proposed method exhibited the highest registration performance compared with existing methods. In particular, when θ is 0.05, an improvement of 22% is observed in the average PCK compared with the SuperGlue method [35].

**Figure 6:**
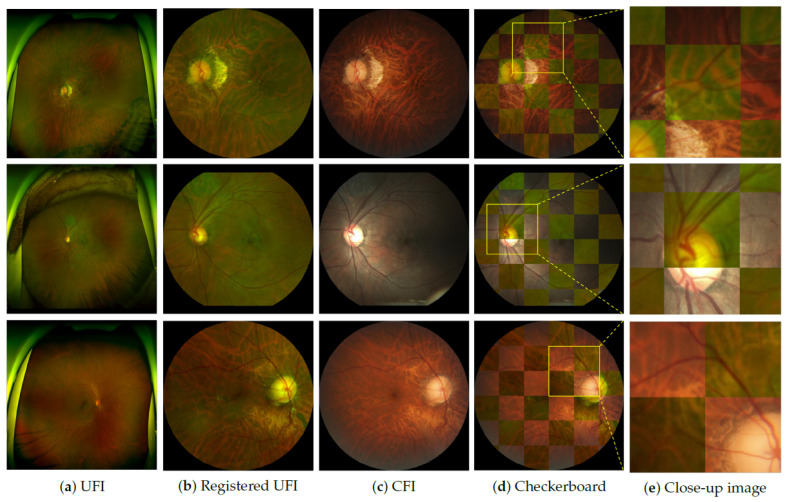
Qualitative results of UFI–CFI registration. Columns show (**a**) original UFI, (**b**) registered UFI, (**c**) corresponding CFI, (**d**) checkerboard overlay for alignment evaluation, and (**e**) close-up of the optic disc region. Checkerboard transitions indicate alignment quality, while close-ups highlight vessel and disc consistency.

Figure 6 shows the qualitative results of the proposed scaled-feature registration method. A common technique for visually assessing the registration accuracy is the checkerboard overlay, which interleaves the registered UFI and target CFI in alternating grid cells. In well-aligned regions, anatomical features such as vessels and OD appear to be continuous across adjacent grid cells. As shown in the first two rows, the proposed method achieved smooth transitions across the grid boundaries, indicating a successful registration. The vascular structures and OD contours were well-aligned, with no noticeable discontinuities. In contrast, the third row reveals minor misalignments, where the vessel segments appear fragmented, or the OD boundary is distorted. These cases are likely to stem from local artifacts or noise in the UFI, leading to partial registration failure. Despite these challenges, the close-up views in the column in Figure 6e demonstrate that most structural features remain well preserved, even in less optimal cases. Overall, the results highlighted the robustness of the proposed feature-based alignment, which effectively preserved anatomical continuity across varying imaging conditions.

### 3.2. Evaluation of UFI-to-CFI Translation

Fundus imaging is crucial for diagnosing various ophthalmic diseases. Age-related macular degeneration (AMD) damages the central retinal area known as the macula and potentially leads to vision loss. Accurately performing the conversion in the macular region during UFI-to-CFI translation is essential. To achieve this, metrics such as mean squared error (MSE), peak signal-to-noise ratio (PSNR), and structural similarity index (SSIM) were used to assess the quality of the translated images [39]. The MSE and PSNR were used to directly compare the differences between image pixels. MSE represents the cumulative squared error between the transformed and original images and PSNR represents the maximum error measurement between the two images. Both metrics measure the overall error in an image, which helps evaluate the translation quality. SSIM measures the similarity between two images using the structural information inherent in the pixels.

The quantitative results of our image translation experiments are summarized in Table 2. A total of 3578 UFI–CFI pairs were used, with 3278 for training and 300 for testing. We benchmarked our proposed method against several leading approaches, including unpaired methods (CycleGAN [7], Yoo et al. [1], Pham et al. [9]) and paired methods (pix2pix [22], pix2pixHD [6]). A clear trend emerged from the results: paired learning strategies consistently outperformed unpaired ones. This trend can likely be attributed to the scaled feature registration in paired methods, which ensures robust alignment between UFI and CFI features while minimizing information loss. Within this strong cohort of paired methods, our proposed model emerged as the top performer. A statistical analysis using paired *t*-tests confirmed that our method achieved significantly better performance than all other comparative models across all evaluation metrics (*p* < 0.001, 95% CI). The key to this superior performance is our novel distorted vessel correction branch. By explicitly preserving fine structural details, this component facilitated more effective training and resulted in higher fidelity image translation.

### 3.3. Qualitative Evaluation for Clinical Usefulness

**Figure 7:**
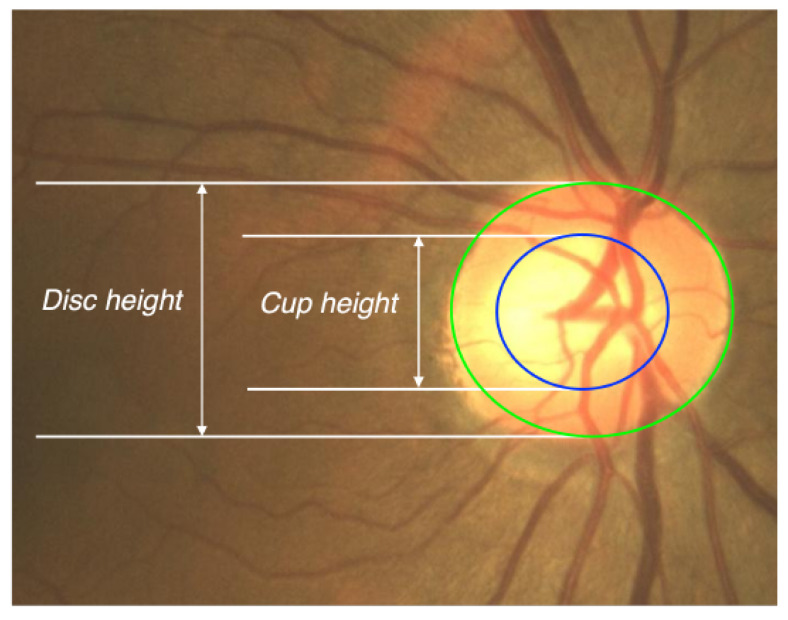
Illustration of the cup-to-disc ratio (CDR) in a fundus image. The green circle outlines the optic disc boundary, and the blue circle indicates the optic cup. The vertical distances labeled as “Disc height” and “Cup height” correspond to the full disc diameter and the inner cup diameter, respectively. The CDR is computed as the ratio between these two vertical measurements, and is a critical clinical indicator for diagnosing and monitoring glaucoma.

To assess clinical usefulness, we conducted a qualitative evaluation focused on features critical for disease assessment, including cup-to-disc and color of the disc characteristics (Figure 7). Two glaucoma specialists independently reviewed 100 test cases in a side-by-side setting, where they compared the reference CFI (ground truth), the registered UFI, and the generated CFI. Three criteria were evaluated: optic nerve structure (cup-to-disc ratio, disc color), vascular distribution (overall vessel morphology, contrast), and drusen (pattern, number). Each item was rated for resemblance to the reference CFI on a four-point scale (3 = good, 2 = moderate, 1 = poor, 0 = not assessable), with higher scores indicating greater similarity. Disagreements were resolved by consensus. For example, a score of 1.53±0.59 for the cup-to-disc ratio on the registered UFI indicates an average resemblance between “poor” and “moderate.”

The quantitative results are summarized in Table 3. Across all criteria, generated CFIs received higher scores than registered UFIs, with differences confirmed as statistically significant by paired *t*-tests (two-sided; all p<0.001; n=100). The ophthalmologists offered the following summary opinion: **“The generated CFIs exhibited such high visual fidelity that they were nearly indistinguishable from real CFIs”**. This evaluation measures translation quality rather than diagnostic performance. Although diagnostic tasks (e.g., diabetic retinopathy or glaucoma grading) were not included, diagnostic agreement studies are planned as future work.

**Figure 8:**
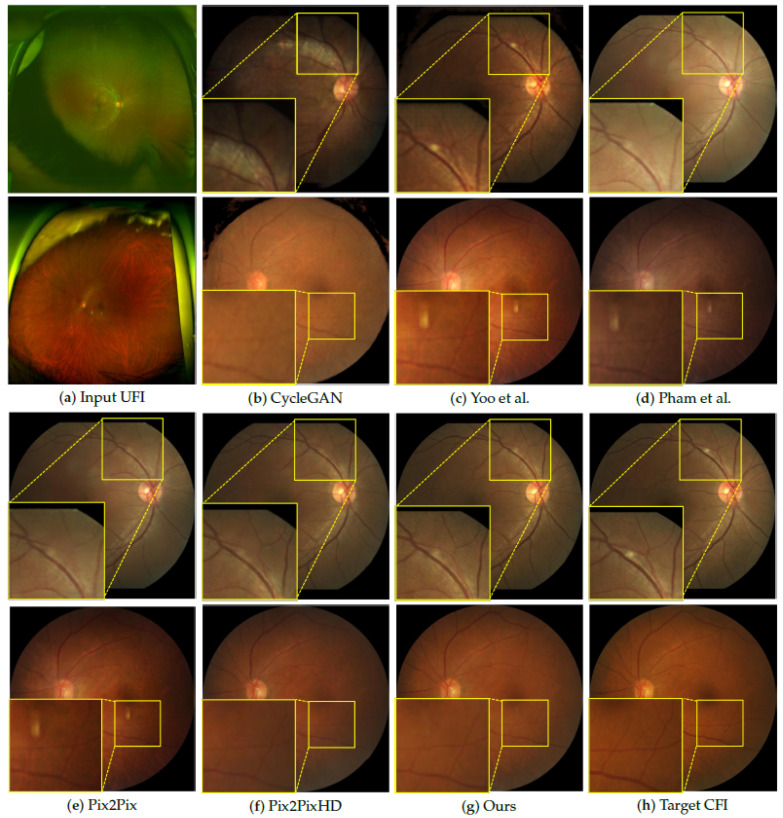
Qualitative comparison of different image translation methods from UFI to CFI. The figure shows the (**a**) input UFI, (**b**) result from CycleGAN [7], (**c**) result from Yoo et al. [1], (**d**) result from Pham et al. [9], (**e**) result from Pix2Pix [22], (**f**) result from Pix2PixHD [6], (**g**) our proposed method, and (**h**) target CFI (ground truth). CycleGAN [7] produces structural distortions, while Yoo et al. [1] and Pham et al. [9] blur fine vascular details. Pix2Pix [22] show relatively strong performance but still lose vessel contrast and fail to fully remove peripheral artifacts. In contrast, the proposed method reconstructs vessels around the optic disc with high fidelity and continuity, and effectively suppresses peripheral artifacts.

Figure 8 shows the qualitative performances of various image-translation methods for converting UFIs into CFIs. The comparison includes the results of CycleGAN [6], Yoo et al. [1], and Pham et al. [7], and the output of the proposed method, all of which were evaluated against the ground-truth CFI. In the first row, which highlights the optic disc region and adjacent vessels, the proposed method demonstrated a marked improvement in vessel depiction. Unlike CycleGAN [6], which induces prominent structural distortions by excessively emphasizing style translation, and Yoo et al. [1] and Pham et al. [7], which tend to blur or smooth fine vascular structures, our approach effectively preserves vessel continuity and clarity. The vessels surrounding the optic disc were reconstructed with high structural fidelity, rendering them similar to the target CFI. These findings demonstrate that the model can maintain anatomically crucial details while ensuring realistic synthesis. The second row focuses on regions prone to peripheral artifacts in UFI. Although Methods (c) and (d) are relatively robust in preserving important anatomical features, they often fail to eliminate the characteristic UFI artifacts and noise patterns, resulting in residual distortions or inconsistent textures. Pix2Pix [22] and Pix2PixHD [6] show relatively strong performance, in reproducing overall structures and achieving stable global color representation; however, they still suffer from local contrast loss and incomplete removal of noise, leading to blurred fine vascular details and color inhomogeneity. In contrast, the proposed method effectively suppresses these artifacts while maintaining a detailed representation of the adjacent vessels. This suggests that the model can disentangle irrelevant pathological image features from structurally relevant anatomies during translation. Overall, while existing approaches either introduce structural distortions, oversmooth fine details, or fail to fully remove peripheral artifacts, the proposed method achieves more accurate and clinically interpretable conversions of UFI to CFI. By preserving the structural fidelity of thin vessel branches in the optic disc region and simultaneously suppressing peripheral noise, our approach produces results that most closely resemble the ground-truth CFI and demonstrate superior clinical applicability compared to competing methods.

### 3.4. Further Experiments

**Computational Performance.** The model was trained on dual NVIDIA RTX A6000 GPUs, reaching a peak memory usage of approximately 72 GB (36 GB per GPU) with a batch size of 64. During inference, the translation step averaged 32 ms per image. The complete pipeline, including both scale adjustment and translation, averaged 52 ms per image, corresponding to a processing speed of approximately 19 frames per second (FPS). This performance demonstrates significant computational efficiency, supporting the model’s feasibility for near-real-time clinical applications.
**External Validataion.** To address the limitations of relying on a single-center dataset, we conducted external validation experiments to assess the generalizability of our model. Specifically, we evaluated its zero-shot generalization capability using public UFI datasets (DeepDRiD, MSHF, and UWF-IQA [40,41,42]) and compared the Fréchet Inception Distance (FID) [43] between real CFIs and the generated CFIs. FID measures the distance between the feature distributions of real and generated images in the latent space of a pre-trained Inception network; lower values indicate closer alignment in terms of both image quality and diversity.

For DeepDRiD and MSHF, FID was computed by comparing the generated CFIs with their corresponding real CFIs. Since UWF-IQA does not provide real CFIs, we instead used the SMC dataset as the reference for comparison. Table 4 summarizes the results, showing that while our method produced plausible CFIs across different datasets, the FID scores indicate that there is still a substantial gap compared with real CFIs. For UWF-IQA, which does not provide real CFIs, we used the SMC dataset as the reference; in this case, the generated CFIs also yielded competitive but non-negligible FID values. These results highlight the challenges of cross-dataset generalization and suggest that further work will be required to optimize performance under varying imaging conditions.

**Figure 9:**
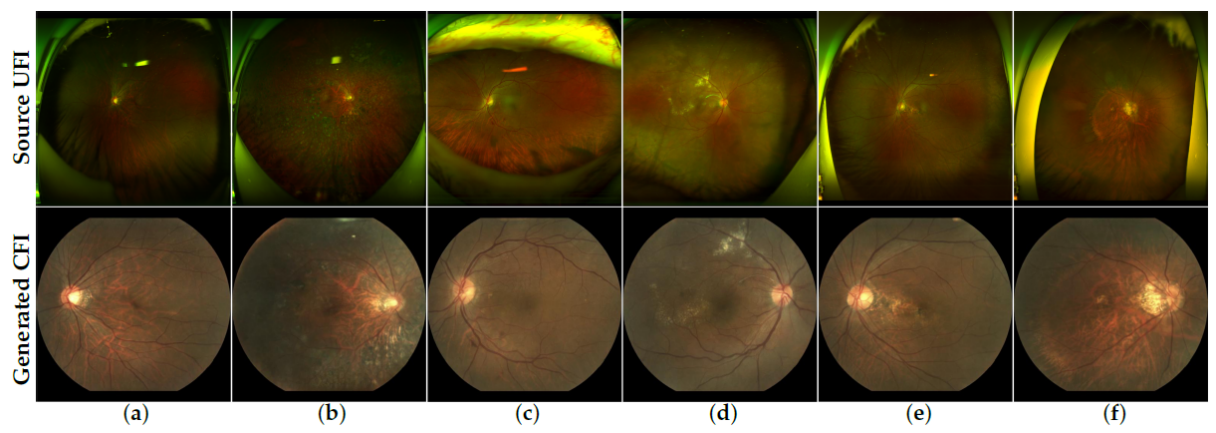
Qualitative results of the proposed UFI-to-CFI translation on various public datasets. The top row displays the source UFI images, while the bottom row shows the corresponding encouraging visual fidelity generated CFI by our model. The dataset order is as follows: (**a**,**b**) DeepDRiD [40], (**c**,**d**) MSHF [41], (**e**,**f**) UWF-IQA [42]; within each pair, the first is OD (right eye) and the second is OS (left eye).

Representative qualitative examples are provided in Figure 9, where the top row shows the source UFIs and the bottom row shows the corresponding generated CFIs. Visual inspection confirms that the generated CFIs preserve overall anatomical structures and vessel morphology across diverse datasets, although subtle differences remain under varying imaging conditions. Taken together, these findings suggest that while our pipeline demonstrates encouraging robustness to unseen datasets, further refinement will be required to fully close the cross-dataset gap.

### 3.5. Ablation Study

Table 5 presents the results of an ablation study designed to isolate the effects of the scaled feature registration and the vessel correction branch. Experiments were conducted on 300 paired images from the SMC fundus dataset, comparing four systematically designed configurations. The baseline configuration, using neither module, exhibited the lowest performance across all metrics (MSE: 70.14, SSIM: 78.74%). Notably, introducing the vessel correction branch without spatial alignment degraded performance relative to the baseline (e.g., SSIM decreased by 5.6% to 74.33%). This finding indicates that vessel correction is fundamentally constrained by misaligned features when anatomical registration is absent. The performance degradation occurs because, without geometric alignment, the branch is supervised by misregistered Laplacian targets, forcing it to learn erroneous edge priors. These noisy priors introduce spatially inconsistent cues into the generator, causing error propagation that results in blurring and vessel-deformation artifacts. Therefore, geometric registration serves as an essential prerequisite for the branch to learn effectively.

Conversely, enabling only scaled feature registration resulted in substantial improvements over the baseline, reducing MSE by 13.5% (to 60.64) and improving SSIM by 11.4% (to 87.71%). These gains underscore the critical importance of spatial normalization for robust image translation. However, alignment alone was insufficient to achieve optimal fidelity. The most pronounced improvements were achieved when both modules were used in synergy. This combination yielded the best performance across all metrics (MSE: 57.38, PSNR: 30.74, SSIM: 88.73%, MS-SSIM: 89.71%), representing a 12.7% SSIM improvement over the baseline. This outcome demonstrates that anatomical alignment provides the necessary foundation, enabling the vessel correction branch to effectively restore fine vascular and optic disc details.

In summary, our ablation analysis revealed that the vessel correction branch is ineffective in isolation and fundamentally relies on the spatial normalization afforded by scaled registration. While registration alone substantially enhanced image quality, maximal fidelity and fine-detail restoration were achieved only through the synergy of both components. This validates the complementary and interdependent roles of each module in our proposed architecture.

## 4. Discussion

Our two-stage approach targets complementary goals: stage 1 suppresses background artifacts and corrects geometric distortions, and stage 2 uses edge-map estimation to refine vascular structures. In a side-by-side review, ophthalmologists observed clearer optic nerve head detail, peripheral branches, and macular features in the generated CFIs than in registered UFIs, suggesting potential clinical utility in a single-center setting. These observations remain preliminary and require prospective, multi-institutional validation.

External testing on public datasets (DeepDRiD, MSHF, UWF-IQA) under a consistent FID protocol suggests that an SMC-trained model can operate beyond its development domain within the limits of distributional metrics. To strengthen generalization, we prioritize (i) multi-institution, multi-vendor validation with task-level endpoints (e.g., eye-disease classification AUC, vessel-segmentation Dice coefficient, lesion-detection AUROC); (ii) lightweight domain adaptation (test-time adaptation, few-shot fine-tuning); and (iii) broader evaluation beyond FID (perceptual metrics and blinded reader studies).

A key limitation is the small paired dataset, which constrains optimization and increases overfitting risk for data-hungry generators. We therefore emphasized data-efficient training (extensive augmentation, patch-based sampling, early stopping, weight decay). The model also tended to struggle with drusen, motivating mechanisms for small, pathology-relevant features (multi-scale attention, pathology-aware losses, higher-resolution inputs). Going forward, we will focus on better separating disease-related structures from multi-scale noise, with practical near-term steps including multi-scale loss designs and anatomy-aware constraints.

Diffusion and transformer generators are promising but presently limited by data and computational demands in our setting. As larger multi-institutional datasets or robust self-supervised pretraining become available, parameter-efficient variants could be integrated (e.g., latent diffusion conditioned on vessels/edges, transformer–UNet hybrids with lightweight adapters).

## 5. Conclusions

We proposed a UFI-to-CFI translation method based on scaled-feature registration and distorted-vessel correction. In expert review, the generated CFIs often appeared closer in visual quality to reference images, in a manner consistent with the quantitative findings, and external testing on public datasets suggested generalization capability beyond our development center. Taken together, these results point to potential clinical applicability in preserving diagnostically relevant anatomy. The evidence remains preliminary. Any clinical deployment should await rigorous, prospective, multi-institutional studies. Planned work includes (i) exploring a hierarchical, region-aware discriminator and (ii) conducting multi-vendor clinical studies to evaluate real-world generalizability. Additionally, we plan a pilot diagnostic agreement study (e.g., DR and glaucoma grading) using generated CFIs to assess task-level utility.

## Figures and Tables

**Table 1:** Quantitative comparison of registration methods.

Methods	Mean PCK (%)		
	θ=0.05	θ=0.1	θ=0.15
SIFT [37]	31.6	46.7	69.0
ORB [38]	27.8	41.9	57.4
SuperGlue [35]	46.9	78.6	89.2
**Ours**	**57.3**	**83.1**	**94.8**

Boldface indicates the best performance at each threshold.

**Table 2:** Comparison of translation methods on the SMC fundus dataset.

Methods		MSE ↓	PSNR ↑	SSIM (%) ↑	MS-SSIM (%) ↑
Unpaired Learning	CycleGAN [7]	90.42	28.60	43.41	46.03
	Yoo et al. [1]	75.78	29.44	75.46	74.87
	Pham et al. [9]	70.19	28.71	78.79	78.74
Paired Learning	Pix2Pix [22]	70.71	29.71	84.49	84.30
	Pix2PixHD [6]	60.64	30.49	87.71	88.02
	Ours	**57.38**	**30.74**	**88.73**	**89.71**

Lower MSE and higher PSNR/SSIM/MS-SSIM indicate better performance (SSIM and MS-SSIM in percent). Arrows indicate the performance change direction: ↑ means improvement, ↓ means degradation. Bold values indicate the highest performance. All improvements of the proposed method over other methods were statistically significant (*p* < 0.001; 95% CI for all comparisons).

**Table 3:** Quality scoring of fundus images was performed by ophthalmologists based on three evaluation criteria, each comprising two items (scored 0–3; higher scores indicate better clinical information quality), to assess the clinical usefulness of the generated CFIs.

Evaluation Criteria	Items	Score (Mean ± SD)	
		Registered UFI (Regi. Only)	Generated CFI (After Trans.)
Optic nerve structures	Cup–to–disc ratio	1.53 ± 0.59	**2.68 ± 0.49**
	Color of the disc	1.23 ± 0.45	**3.00 ± 0.00**
Vascular distribution	Overall morphology	2.43 ± 0.50	**2.83 ± 0.38**
	Vessel contrast	1.87 ± 0.63	**2.66 ± 0.50**
Drusen	Drusen pattern	2.18 ± 0.41	**2.53 ± 0.50**
	Drusen number	2.24 ± 0.43	**2.53 ± 0.50**

Regi. is registration; Trans. is translation; Boldface values denote statistically higher scores for generated CFI compared with registerd UFI (*p* < 0.001; *n* = 300).

**Table 4:** FID scores for external validation on public UFI datasets.

Dataset	Comparison	FID ↓
UWF-IQA [42]	generated CFI vs. SMC real CFI	51.72
MSHF [41]	generated CFI vs. real CFI	104.32
DeepDRiD [40]	generated CFI vs. real CFI	132.24

Arrows indicate the performance change direction: ↑ means improvement, ↓ means degradation. All comparisons were statistically significant under the same evaluation protocol (two-sided tests; *p* < 0.001).

**Table 5:** Ablation study on the effects of scaled feature registration and vessel correction branch in UFI-to-CFI translation.

Method Components		Performance Metrics			
Scaled Feature Registration	Vessel Correction Registration	MSE	PSNR	SSIM (%)	MS-SSIM (%)
		↓	↑	↑	↑
✗	✗	70.14	28.67	78.74	79.63
✗	✓	78.96	28.04	74.33	73.91
✓	✗	60.64	30.49	87.71	88.02
✓	✓	**57.38**	**30.74**	**88.73**	**89.71**

Lower MSE and higher PSNR/SSIM/MS-SSIM denote better performance; Arrows indicate the performance change direction: ↑ means improvement, ↓ means degradation. bold marks the best per metric.

## Data Availability

The datasets presented in this article are not readily available due to hospital privacy regulations and as they were collected for a retrospective clinical study. Requests to access the datasets should be directed to the corresponding author.

## Abbreviations

The following abbreviations are used in this manuscript:

CFI	Conventional Fundus Image
UFI	Ultrawidefield Fundus Image
OD	Optic Disc
AMD	Age-related Macular Degeneration
ERM	Epiretinal Membranes
CNN	Convolutional Neural Network
GNN	Graph-base Neural Network
cGAN	conditional Generative Adversarial Networks
CBAM	Convolutional Block Attention Module
PCK	Percentage of Correct Key points
MSE	Mean Squared Error
PSNR	Peak Signal-to-Noise Ratio
SSIM	Structural Similarity Index
MS-SSIM	Multiscale Structural Similarity Index

## References

[1] Yoo T.K., Ryu I.H., Kim J.K., Lee I.S., Kim J.S., Kim H.K., Choi J.Y. (2020). Deep learning can generate traditional retinal fundus photographs using ultrawidefield images via generative adversarial networks. Comput. Methods Programs Biomed..

[2] Peng Y., Dharssi S., Chen Q., Keenan T.D., Agrón E., Wong W.T., Chew E.Y., Lu Z. (2019). DeepSeeNet: A deep learning model for automated classification of patient-based age-related macular degeneration severity from color fundus photographs. Ophthalmology.

[3] Patel S.N., Shi A., Wibbelsman T.D., Klufas M.A. (2020). Ultra-widefield retinal imaging: An update on recent advances. Ther. Adv. Ophthalmol..

[4] Nagiel A., Lalane R.A., Sadda S.R., Schwartz S.D. (2016). Ultra-widefield fundus imaging: A review of clinical applications and future trends. Retina.

[5] Lee J.A., Liu P., Cheng J., Fu H. A deep step pattern representation for multimodal retinal image registration. Proceedings of the IEEE/CVF International Conference on Computer Vision.

[6] Wang T.C., Liu M.Y., Zhu J.Y., Tao A., Kautz J., Catanzaro B. High-resolution image synthesis and semantic manipulation with conditional GANs. Proceedings of the IEEE Conference on Computer Vision and Pattern Recognition.

[7] Zhu J.Y., Park T., Isola P., Efros A.A. Unpaired Image-to-Image Translation using Cycle-Consistent Adversarial Networks. Proceedings of the IEEE International Conference on Computer Vision.

[8] Perez-Rovira A., Trucco E., Wilson P., Liu J. Deformable registration of retinal fluorescein angiogram sequences using vasculature structures. Proceedings of the 2010 Annual International Conference of the IEEE Engineering in Medicine and Biology.

[9] Pham V.-N., Le D.T., Bum J., Lee E.J., Han J.C., Choo H. (2023). Attention-aided generative learning for multi-scale multi-modal fundus image translation. IEEE Access.

[10] Zhang Q., Nie Y., Zheng W.S. (2019). Dual illumination estimation for robust exposure correction. Comput. Graph. Forum.

[11] Woo S., Park J., Lee J.Y., Kweon I.S. CBAM: Convolutional block attention module. Proceedings of the European Conference on Computer Vision (ECCV).

[12] Ren S., He K., Girshick R., Sun J. (2016). Faster R-CNN: Towards real-time object detection with region proposal networks. IEEE Trans. Pattern Anal. Mach. Intell..

[13] Tu H., Wang Z., Zhao Y. (2024). Unpaired image-to-image translation with diffusion adversarial network. Mathematics.

[14] Go S., Ji Y., Park S.J., Lee S. Generation of structurally realistic retinal fundus images with diffusion models. Proceedings of the IEEE/CVF Conference on Computer Vision and Pattern Recognition (CVPR) Workshops.

[15] Zhang L., Rao A., Agrawala M. Adding conditional control to text-to-image diffusion models. Proceedings of the IEEE/CVF International Conference on Computer Vision.

[16] Fang Z., Chen Z., Wei P., Li W., Zhang S., Elazab A., Jia G., Ge R., Wang C. UWAT-GAN: Fundus fluorescein angiography synthesis via ultra-wide-angle transformation multi-scale GAN. Proceedings of the 26th International Conference on Medical Image Computing and Computer-Assisted Intervention (MICCAI).

[17] Ge R., Fang Z., Wei P., Chen Z., Jiang H., Elazab A., Li W., Wan X., Zhang S., Wang C. (2024). UWAFA-GAN: Ultra-wide-angle fluorescein angiography transformation via multi-scale generation and registration enhancement. IEEE J. Biomed. Health Inform..

[18] Wang D., Lian J., Jiao W. (2024). Multi-label classification of retinal disease via a novel vision transformer model. Front. Neurosci..

[19] Malik S.M.F., Nafis M.T., Ahad M.A., Tanweer S. (2024). Grading and Anomaly Detection for Automated Retinal Image Analysis using Deep Learning. arXiv.

[20] Wang Y. Research on the application of vision transformer model in 14-visual-field classification of fundus images. Proceedings of the International Conference on Computer Application and Information Security (ICCAIS 2024).

[21] Huang C., Jiang Y., Yang X., Wei C., Chen H., Xiong W., Lin H., Wang X., Tian T., Tan H. (2024). Enhancing Retinal Fundus Image Quality Assessment with Swin-Transformer–Based Learning Across Multiple Color-Spaces. Transl. Vis. Sci. Technol..

[22] Isola P., Zhu J.Y., Zhou T., Efros A.A. Image-to-image translation with conditional adversarial networks. Proceedings of the IEEE Conference on Computer Vision and Pattern Recognition.

[23] Lee M.E., Kim S.H., Seo I.H. Intensity-based registration of medical images. Proceedings of the 2009 International Conference on Test and Measurement.

[24] Luo G., Chen X., Shi F., Peng Y., Xiang D., Chen Q., Xu X., Zhu W., Fan Y. (2020). Multimodal affine registration for ICGA and MCSL fundus images of high myopia. Biomed. Opt. Express.

[25] Hernandez M., Medioni G., Hu Z., Sadda S. Multimodal registration of multiple retinal images based on line structures. Proceedings of the 2015 IEEE Winter Conference on Applications of Computer Vision.

[26] Li Z., Huang F., Zhang J., Dashtbozorg B., Abbasi-Sureshjani S., Sun Y., Long X., Yu Q., Romeny B.t.H., Tan T. (2018). Multi-modal and multi-vendor retina image registration. Biomed. Opt. Express.

[27] Hervella Á.S., Rouco J., Novo J., Ortega M. (2018). Multimodal registration of retinal images using domain-specific landmarks and vessel enhancement. Proc. Comput. Sci..

[28] Ghassabi Z., Shanbehzadeh J., Sedaghat A., Fatemizadeh E. (2013). An efficient approach for robust multimodal retinal image registration based on UR-SIFT Features and PIIFD descriptors. EURASIP J. Image Video Process..

[29] Wang G., Wang Z., Chen Y., Zhao W. (2015). Robust point matching method for multimodal retinal image registration. Biomed. Signal Process. Control.

[30] Chen J., Tian J., Lee N., Zheng J., Smith R.T., Laine A.F. (2010). A partial intensity invariant feature descriptor for multimodal retinal image registration. IEEE Trans. Biomed. Eng..

[31] Lee J.A., Cheng J., Lee B.H., Ong E.P., Xu G., Wong D.W.K., Liu J., Laude A., Lim T.H. A low-dimensional step pattern analysis algorithm with application to multimodal retinal image registration. Proceedings of the IEEE Conference on Computer Vision and Pattern Recognition (CVPR).

[32] Rocco I., Arandjelović R., Sivic J. Convolutional neural network architecture for geometric matching. Proceedings of the IEEE Conference on Computer Vision and Pattern Recognition.

[33] Ballé J., Chou P.A., Minnen D., Singh S., Johnston N., Agustsson E., Hwang S.J., Toderici G. (2020). Nonlinear transform coding. IEEE J. Sel. Top. Signal Process..

[34] Li H., Liu H., Hu Y., Higashita R., Zhao Y., Qi H., Liu J. Restoration of cataract fundus images via unsupervised domain adaptation. Proceedings of the 2021 IEEE 18th International Symposium on Biomedical Imaging (ISBI).

[35] Sarlin P.E., DeTone D., Malisiewicz T., Rabinovich A. SuperGlue: Learning feature matching with graph neural networks. Proceedings of the IEEE/CVF Conference on Computer Vision and Pattern Recognition.

[36] Lyu W., Chen L., Zhou Z., Wu W. (2021). Weakly supervised object-aware convolutional neural networks for semantic feature matching. Neurocomputing.

[37] Lowe D.G. (2004). Distinctive image features from scale-invariant keypoints. Int. J. Comput. Vis..

[38] Rublee E., Rabaud V., Konolige K., Bradski G. ORB: An efficient alternative to SIFT or SURF. Proceedings of the 2011 International Conference on Computer Vision.

[39] Sara U., Akter M., Uddin M.S. (2019). Image quality assessment through FSIM, SSIM, MSE and PSNR—A Comparative Study. J. Comput. Commun..

[40] Liu R., Wang X., Wu Q., Dai L., Fang X., Yan T., Son J., Tang S., Li J., Gao Z. (2022). DeepDRiD: Diabetic retinopathy—grading and image quality estimation challenge. Patterns.

[41] He S., Ye X., Xie W., Shen Y., Yang S., Zhong X., Guan H., Zhou X., Wu J., Shen L. (2024). Open ultrawidefield fundus image dataset with disease diagnosis and clinical image quality assessment. Sci. Data.

[42] Jin K., Gao Z., Jiang X., Wang Y., Ma X., Li Y., Ye J. (2023). MSHF: A Multi-Source Heterogeneous Fundus (MSHF) Dataset for Image Quality Assessment. Sci. Data.

[43] Heusel M., Ramsauer H., Unterthiner T., Nessler B., Hochreiter S. (2017). GANs Trained by a Two Time-Scale Update Rule Converge to a Local Nash Equilibrium. Adv. Neural Inf. Process. Syst..

